# 2-Chloro-4-nitro­benzoic acid–quinoline (1/1)

**DOI:** 10.1107/S160053681104075X

**Published:** 2011-10-08

**Authors:** Kazuma Gotoh, Hiroyuki Ishida

**Affiliations:** a Department of Chemistry, Faculty of Science, Okayama University, Okayama 700-8530, Japan

## Abstract

In the title compound, C_7_H_4_ClNO_4_·C_9_H_7_N, the two components are connected by an O—H⋯N hydrogen bond. In the hydrogen-bonded unit, the dihedral angle between the quinoline ring system and the benzene ring of benzoic acid is 3.15 (7)°. In the crystal, units are linked by inter­molecular C—H⋯O hydrogen bonds, forming a tape along the *c* axis. The tapes are stacked along the *b* axis through a C—H⋯O hydrogen bond into a layer parallel to the *bc* plane.

## Related literature

For related structures, see: Gotoh & Ishida (2009 ▶); Gotoh *et al.* (2010 ▶).
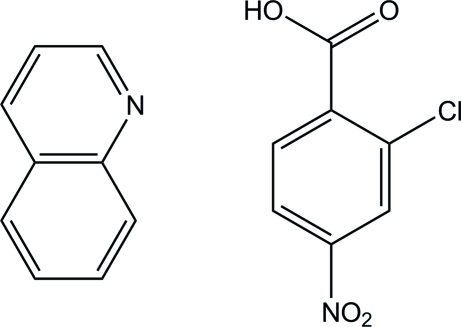


## Experimental

### 

#### Crystal data

C_9_H_7_N·C_7_H_4_ClNO_4_
*M* *_r_* = 330.73
Orthorhombic, 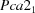

*a* = 31.125 (3) Å
*b* = 3.7560 (3) Å
*c* = 12.3615 (12) Å
*V* = 1445.1 (2) Å^3^
*Z* = 4
Mo *K*α radiation
μ = 0.29 mm^−1^
*T* = 185 K
0.35 × 0.22 × 0.06 mm

#### Data collection

Rigaku R-AXIS RAPID II diffractometer
Absorption correction: numerical (*NUMABS*; Higashi, 1999 ▶) *T*_min_ = 0.925, *T*_max_ = 0.983
17044 measured reflections
4166 independent reflections
3681 reflections with *I* > 2σ(*I*)
*R*_int_ = 0.053

#### Refinement

*R*[*F* ^2^ > 2σ(*F* ^2^)] = 0.042
*wR*(*F* ^2^) = 0.083
*S* = 1.06
4166 reflections
212 parameters
1 restraint
H atoms treated by a mixture of independent and constrained refinement
Δρ_max_ = 0.23 e Å^−3^
Δρ_min_ = −0.25 e Å^−3^
Absolute structure: Flack (1983 ▶), 1989 Friedel pairs
Flack parameter: 0.01 (5)

### 

Data collection: *PROCESS-AUTO* (Rigaku/MSC, 2004 ▶); cell refinement: *PROCESS-AUTO*; data reduction: *CrystalStructure* (Rigaku/MSC, 2004 ▶); program(s) used to solve structure: *SHELXS97* (Sheldrick, 2008 ▶); program(s) used to refine structure: *SHELXL97* (Sheldrick, 2008 ▶); molecular graphics: *ORTEP-3* (Farrugia, 1997) ▶; software used to prepare material for publication: *CrystalStructure* and *PLATON* (Spek, 2009 ▶).

## Supplementary Material

Crystal structure: contains datablock(s) global, I. DOI: 10.1107/S160053681104075X/lh5345sup1.cif

Structure factors: contains datablock(s) I. DOI: 10.1107/S160053681104075X/lh5345Isup2.hkl

Supplementary material file. DOI: 10.1107/S160053681104075X/lh5345Isup3.cml

Additional supplementary materials:  crystallographic information; 3D view; checkCIF report

## Figures and Tables

**Table 1:** Hydrogen-bond geometry (Å, °)

*D*—H⋯*A*	*D*—H	H⋯*A*	*D*⋯*A*	*D*—H⋯*A*
O1—H1⋯N2	0.95 (3)	1.65 (3)	2.595 (2)	177 (3)
C5—H5⋯O2^i^	0.95	2.44	3.251 (2)	143
C9—H9⋯O4^ii^	0.95	2.57	3.365 (3)	141
C14—H14⋯O3^i^	0.95	2.55	3.476 (3)	165

Symmetry codes: (i) 
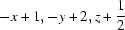
; (ii) 
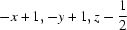
.

**Fig. 1.**
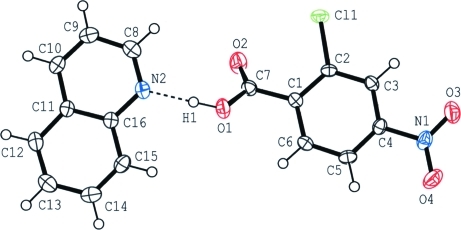
The molecular structure of the title compound. Displacement ellipsoids of non-H atoms are drawn at the 50% probability level. The dashed line indicates the O—H···N hydrogen bond.

**Fig. 2.**
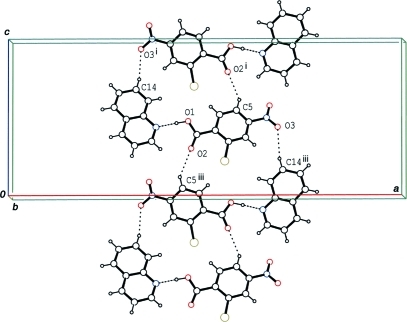
A partial packing diagram of the title compound viewed along the b axis, showing a layer parallel to the bc plane formed by O—H···N and C—H···O hydrogen bonds (dashed lines). [Symmetry codes: (i) -x + 1, -y + 2, z + 1/2; (iii) -x + 1, -y + 2, z - 1/2.]

## supplementary crystallographic information

### Comment

The title compound was prepared in order to extend our study on *D*—H···*A* hydrogen bonding (*D* = N, O, or C; *A* = N, O or Cl) in quinoline–substituted benzoic acid systems (Gotoh & Ishida, 2009; Gotoh *et al.*, 2010).

In the crystal structure of the title compound, no acid-base interaction involving proton transfer is observed between the two components, which are linked by an O—H···N hydrogen bond (Table 1 and Fig. 1). In the hydrogen-bonded unit, the dihedral angle between the quinoline ring system and the benzene ring of the benzoic acid is 3.15 (7)°. The carboxyl plane makes dihedral angles of 43.0 (2) and 39.9 (2)°, respectively, with the quinoline ring system and the benzene ring. The two components are further linked by intermolecular C—H···O hydrogen bonds (Table 1), forming a tape along the *c* axis and the tapes are stacked along the *b* axis through an C—H···O hydrogen bond into a layer parallel to the *bc* plane (Fig. 2). No significant interaction is observed between the layers.

### Experimental

Single crystals were obtained by slow evaporation from an acetonitrile solution (30 ml) of 2-chloro-4-nitrobenzoic acid (233 mg) and quinoline (150 mg) at room temperature.

### Refinement

C-bound H atoms were positioned geometrically (C—H = 0.95 Å) and refined as riding, with *U*_iso_(H) = 1.2*U*_eq_(C). The O-bound H atom was found in a difference Fourier map and refined freely. The refined O—H distance is 0.95 (3) Å. Flack and Hooft parameters are 0.01 (5) and 0.02 (3), respectively.

### Crystal data

**Table:**

C_9_H_7_N·C_7_H_4_ClNO_4_	*F*(000) = 680.00
*M**_r_* = 330.73	*D*_x_ = 1.520 Mg m^−^^3^
Orthorhombic, *P**c**a*2_1_	Mo *K*α radiation, λ = 0.71075 Å
Hall symbol: P 2c -2ac	Cell parameters from 13096 reflections
*a* = 31.125 (3) Å	θ = 3.1–29.9°
*b* = 3.7560 (3) Å	µ = 0.29 mm^−^^1^
*c* = 12.3615 (12) Å	*T* = 185 K
*V* = 1445.1 (2) Å^3^	Platelet, colorless
*Z* = 4	0.35 × 0.22 × 0.06 mm

### Data collection

**Table:**

Rigaku R-AXIS RAPID II diffractometer	3681 reflections with *I* > 2σ(*I*)
Detector resolution: 10.00 pixels mm^-1^	*R*_int_ = 0.053
ω scans	θ_max_ = 29.9°, θ_min_ = 3.1°
Absorption correction: numerical (*NUMABS*; Higashi, 1999)	*h* = −42→43
*T*_min_ = 0.925, *T*_max_ = 0.983	*k* = −4→5
17044 measured reflections	*l* = −17→17
4166 independent reflections	

### Refinement

**Table:**

Refinement on *F*^2^	Secondary atom site location: difference Fourier map
Least-squares matrix: full	Hydrogen site location: inferred from neighbouring sites
*R*[*F*^2^ > 2σ(*F*^2^)] = 0.042	H atoms treated by a mixture of independent and constrained refinement
*wR*(*F*^2^) = 0.083	*w* = 1/[σ^2^(*F*_o_^2^) + (0.0315*P*)^2^ + 0.3405*P*] where *P* = (*F*_o_^2^ + 2*F*_c_^2^)/3
*S* = 1.06	(Δ/σ)_max_ = 0.002
4166 reflections	Δρ_max_ = 0.23 e Å^−^^3^
212 parameters	Δρ_min_ = −0.25 e Å^−^^3^
1 restraint	Absolute structure: Flack (1983), 1989 Friedel pairs
Primary atom site location: structure-invariant direct methods	Flack parameter: 0.01 (5)

### Special details

**Table:**

Geometry. All e.s.d.'s (except the e.s.d. in the dihedral angle between two l.s. planes) are estimated using the full covariance matrix. The cell e.s.d.'s are taken into account individually in the estimation of e.s.d.'s in distances, angles and torsion angles; correlations between e.s.d.'s in cell parameters are only used when they are defined by crystal symmetry. An approximate (isotropic) treatment of cell e.s.d.'s is used for estimating e.s.d.'s involving l.s. planes.
Refinement. Refinement of *F*^2^ against ALL reflections. The weighted *R*-factor *wR* and goodness of fit *S* are based on *F*^2^, conventional *R*-factors *R* are based on *F*, with *F* set to zero for negative *F*^2^. The threshold expression of *F*^2^ > σ(*F*^2^) is used only for calculating *R*-factors(gt) *etc*. and is not relevant to the choice of reflections for refinement. *R*-factors based on *F*^2^ are statistically about twice as large as those based on *F*, and *R*- factors based on ALL data will be even larger.

### Fractional atomic coordinates and isotropic or equivalent isotropic displacement parameters (Å^2^)

**Table:**

	*x*	*y*	*z*	*U*_iso_*/*U*_eq_	
Cl1	0.543417 (13)	0.59426 (12)	0.23410 (4)	0.02675 (10)	
O1	0.45038 (4)	0.5860 (4)	0.48865 (12)	0.0318 (3)	
O2	0.45582 (4)	0.8025 (5)	0.32051 (12)	0.0388 (4)	
O3	0.67381 (4)	0.9928 (5)	0.46192 (13)	0.0456 (4)	
O4	0.65022 (5)	1.2634 (5)	0.60477 (13)	0.0426 (4)	
N1	0.64473 (5)	1.0872 (5)	0.52256 (13)	0.0292 (4)	
N2	0.37038 (5)	0.4475 (4)	0.44710 (14)	0.0263 (3)	
C1	0.51745 (5)	0.8089 (5)	0.43578 (14)	0.0201 (3)	
C2	0.55126 (5)	0.7634 (5)	0.36266 (14)	0.0193 (3)	
C3	0.59320 (5)	0.8484 (5)	0.39161 (14)	0.0215 (3)	
H3	0.6163	0.8124	0.3427	0.026*	
C4	0.60026 (5)	0.9870 (5)	0.49371 (15)	0.0219 (3)	
C5	0.56809 (6)	1.0347 (5)	0.56935 (14)	0.0238 (4)	
H5	0.5741	1.1294	0.6390	0.029*	
C6	0.52678 (6)	0.9388 (5)	0.53943 (14)	0.0230 (4)	
H6	0.5042	0.9618	0.5906	0.028*	
C7	0.47115 (6)	0.7299 (5)	0.40757 (15)	0.0237 (4)	
C8	0.36085 (7)	0.3048 (5)	0.35296 (17)	0.0303 (4)	
H8	0.3834	0.2672	0.3024	0.036*	
C9	0.31878 (7)	0.2050 (5)	0.32333 (16)	0.0312 (4)	
H9	0.3132	0.1036	0.2543	0.037*	
C10	0.28633 (6)	0.2568 (5)	0.39549 (16)	0.0276 (4)	
H10	0.2579	0.1874	0.3775	0.033*	
C11	0.29493 (5)	0.4134 (5)	0.49705 (15)	0.0223 (4)	
C12	0.26293 (6)	0.4842 (5)	0.57556 (16)	0.0280 (4)	
H12	0.2338	0.4248	0.5610	0.034*	
C13	0.27357 (7)	0.6361 (5)	0.67138 (17)	0.0322 (4)	
H13	0.2518	0.6830	0.7232	0.039*	
C14	0.31654 (7)	0.7255 (5)	0.69516 (18)	0.0325 (4)	
H14	0.3235	0.8291	0.7631	0.039*	
C15	0.34829 (6)	0.6641 (5)	0.62122 (16)	0.0280 (4)	
H15	0.3771	0.7274	0.6375	0.034*	
C16	0.33828 (5)	0.5067 (5)	0.52058 (14)	0.0216 (4)	
H1	0.4212 (11)	0.530 (9)	0.475 (3)	0.092 (11)*	

### Atomic displacement parameters (Å^2^)

**Table:**

	*U*^11^	*U*^22^	*U*^33^	*U*^12^	*U*^13^	*U*^23^
Cl1	0.03042 (19)	0.0318 (2)	0.01799 (17)	0.00371 (18)	−0.00209 (19)	−0.00441 (19)
O1	0.0202 (6)	0.0471 (9)	0.0279 (7)	−0.0040 (6)	0.0009 (5)	0.0086 (7)
O2	0.0261 (6)	0.0614 (10)	0.0289 (8)	−0.0008 (7)	−0.0046 (6)	0.0121 (8)
O3	0.0224 (6)	0.0755 (12)	0.0389 (9)	−0.0026 (7)	0.0008 (6)	0.0006 (8)
O4	0.0397 (8)	0.0547 (10)	0.0332 (8)	−0.0134 (8)	−0.0120 (7)	−0.0041 (7)
N1	0.0250 (7)	0.0365 (10)	0.0261 (8)	−0.0057 (7)	−0.0072 (6)	0.0073 (7)
N2	0.0203 (7)	0.0288 (8)	0.0298 (8)	0.0012 (6)	0.0013 (6)	0.0033 (7)
C1	0.0207 (8)	0.0200 (8)	0.0197 (8)	0.0023 (6)	0.0009 (6)	0.0028 (7)
C2	0.0249 (8)	0.0183 (8)	0.0148 (7)	0.0020 (7)	−0.0011 (6)	−0.0005 (6)
C3	0.0198 (7)	0.0256 (9)	0.0191 (8)	0.0023 (7)	0.0025 (6)	0.0032 (7)
C4	0.0205 (7)	0.0230 (8)	0.0222 (8)	−0.0011 (7)	−0.0039 (6)	0.0031 (7)
C5	0.0316 (9)	0.0224 (9)	0.0175 (8)	0.0014 (7)	−0.0028 (7)	0.0001 (7)
C6	0.0248 (8)	0.0253 (10)	0.0191 (8)	0.0010 (7)	0.0039 (6)	0.0010 (7)
C7	0.0225 (8)	0.0278 (10)	0.0209 (9)	0.0019 (7)	0.0005 (7)	0.0015 (7)
C8	0.0316 (9)	0.0298 (10)	0.0295 (10)	0.0043 (8)	0.0055 (8)	0.0010 (8)
C9	0.0392 (10)	0.0291 (10)	0.0254 (10)	−0.0019 (8)	−0.0026 (8)	−0.0027 (8)
C10	0.0264 (9)	0.0254 (9)	0.0309 (10)	−0.0042 (8)	−0.0049 (8)	0.0006 (8)
C11	0.0207 (7)	0.0193 (9)	0.0269 (9)	0.0014 (6)	−0.0015 (7)	0.0023 (7)
C12	0.0221 (8)	0.0240 (9)	0.0380 (11)	0.0002 (7)	0.0036 (7)	0.0029 (9)
C13	0.0356 (10)	0.0273 (10)	0.0337 (11)	0.0023 (8)	0.0104 (8)	0.0011 (8)
C14	0.0455 (11)	0.0268 (10)	0.0252 (9)	−0.0019 (9)	−0.0012 (9)	−0.0018 (8)
C15	0.0274 (9)	0.0285 (10)	0.0282 (10)	−0.0032 (8)	−0.0076 (7)	0.0032 (8)
C16	0.0214 (7)	0.0187 (8)	0.0248 (9)	0.0011 (6)	−0.0009 (6)	0.0029 (7)

### Geometric parameters (Å, °)

**Table:**

Cl1—C2	1.7288 (18)	C6—H6	0.9500
O1—C7	1.309 (2)	C8—C9	1.411 (3)
O1—H1	0.95 (4)	C8—H8	0.9500
O2—C7	1.208 (2)	C9—C10	1.361 (3)
O3—N1	1.228 (2)	C9—H9	0.9500
O4—N1	1.225 (2)	C10—C11	1.412 (3)
N1—C4	1.478 (2)	C10—H10	0.9500
N2—C8	1.315 (3)	C11—C12	1.416 (2)
N2—C16	1.368 (2)	C11—C16	1.424 (2)
C1—C2	1.398 (2)	C12—C13	1.356 (3)
C1—C6	1.402 (2)	C12—H12	0.9500
C1—C7	1.512 (2)	C13—C14	1.410 (3)
C2—C3	1.391 (2)	C13—H13	0.9500
C3—C4	1.383 (3)	C14—C15	1.366 (3)
C3—H3	0.9500	C14—H14	0.9500
C4—C5	1.382 (2)	C15—C16	1.412 (3)
C5—C6	1.386 (3)	C15—H15	0.9500
C5—H5	0.9500		
			
C7—O1—H1	115 (2)	N2—C8—H8	118.4
O4—N1—O3	124.05 (16)	C9—C8—H8	118.4
O4—N1—C4	117.93 (16)	C10—C9—C8	118.72 (19)
O3—N1—C4	118.01 (17)	C10—C9—H9	120.6
C8—N2—C16	119.27 (16)	C8—C9—H9	120.6
C2—C1—C6	118.54 (16)	C9—C10—C11	120.10 (18)
C2—C1—C7	122.99 (16)	C9—C10—H10	119.9
C6—C1—C7	118.46 (15)	C11—C10—H10	119.9
C3—C2—C1	120.81 (16)	C10—C11—C12	123.67 (16)
C3—C2—Cl1	116.97 (13)	C10—C11—C16	117.62 (16)
C1—C2—Cl1	122.21 (13)	C12—C11—C16	118.71 (17)
C4—C3—C2	118.06 (16)	C13—C12—C11	120.40 (17)
C4—C3—H3	121.0	C13—C12—H12	119.8
C2—C3—H3	121.0	C11—C12—H12	119.8
C5—C4—C3	123.47 (16)	C12—C13—C14	120.93 (19)
C5—C4—N1	118.84 (16)	C12—C13—H13	119.5
C3—C4—N1	117.70 (16)	C14—C13—H13	119.5
C4—C5—C6	117.27 (16)	C15—C14—C13	120.44 (19)
C4—C5—H5	121.4	C15—C14—H14	119.8
C6—C5—H5	121.4	C13—C14—H14	119.8
C5—C6—C1	121.79 (16)	C14—C15—C16	120.04 (18)
C5—C6—H6	119.1	C14—C15—H15	120.0
C1—C6—H6	119.1	C16—C15—H15	120.0
O2—C7—O1	125.46 (17)	N2—C16—C15	119.44 (16)
O2—C7—C1	122.49 (17)	N2—C16—C11	121.08 (16)
O1—C7—C1	112.03 (15)	C15—C16—C11	119.48 (16)
N2—C8—C9	123.19 (18)		
			
C6—C1—C2—C3	−0.6 (3)	C6—C1—C7—O1	−39.1 (2)
C7—C1—C2—C3	178.35 (17)	C16—N2—C8—C9	−0.6 (3)
C6—C1—C2—Cl1	178.32 (14)	N2—C8—C9—C10	−0.3 (3)
C7—C1—C2—Cl1	−2.7 (2)	C8—C9—C10—C11	1.1 (3)
C1—C2—C3—C4	−1.5 (3)	C9—C10—C11—C12	178.46 (18)
Cl1—C2—C3—C4	179.46 (13)	C9—C10—C11—C16	−1.1 (3)
C2—C3—C4—C5	2.1 (3)	C10—C11—C12—C13	−179.86 (18)
C2—C3—C4—N1	−178.12 (16)	C16—C11—C12—C13	−0.3 (3)
O4—N1—C4—C5	−12.2 (3)	C11—C12—C13—C14	−0.3 (3)
O3—N1—C4—C5	168.76 (18)	C12—C13—C14—C15	0.8 (3)
O4—N1—C4—C3	168.02 (18)	C13—C14—C15—C16	−0.7 (3)
O3—N1—C4—C3	−11.0 (3)	C8—N2—C16—C15	−179.17 (18)
C3—C4—C5—C6	−0.4 (3)	C8—N2—C16—C11	0.6 (3)
N1—C4—C5—C6	179.82 (17)	C14—C15—C16—N2	179.84 (18)
C4—C5—C6—C1	−1.9 (3)	C14—C15—C16—C11	0.1 (3)
C2—C1—C6—C5	2.4 (3)	C10—C11—C16—N2	0.2 (3)
C7—C1—C6—C5	−176.60 (18)	C12—C11—C16—N2	−179.33 (16)
C2—C1—C7—O2	−39.8 (3)	C10—C11—C16—C15	179.98 (17)
C6—C1—C7—O2	139.2 (2)	C12—C11—C16—C15	0.4 (3)
C2—C1—C7—O1	141.92 (18)		

### Hydrogen-bond geometry (Å, °)

**Table:**

*D*—H···*A*	*D*—H	H···*A*	*D*···*A*	*D*—H···*A*
O1—H1···N2	0.95 (3)	1.65 (3)	2.595 (2)	177 (3)
C5—H5···O2^i^	0.95	2.44	3.251 (2)	143
C9—H9···O4^ii^	0.95	2.57	3.365 (3)	141
C14—H14···O3^i^	0.95	2.55	3.476 (3)	165

Symmetry codes: (i) −*x*+1, −*y*+2, *z*+1/2; (ii) −*x*+1, −*y*+1, *z*−1/2.
